# The Paediatric Intensive Care Adaptive Platform Trial (PIVOTAL): Turning Pediatric Critical Care Practice Into Clinical Research

**DOI:** 10.1097/PCC.0000000000003937

**Published:** 2026-03-23

**Authors:** Mark J. Peters, Paul Mouncey, Alexina Mason, Tasnin Shahid, Padmanabhan Ramnarayan, Samiran Ray, Katherine Brown, Irene Chang, Doug W. Gould, David Harrison, and the Paediatric Intensive Care Adaptive Platform Trial (PIVOTAL) investigators for Paediatric Critical Care Society Study Group (PCCS-SG), Nazima Pathan

**Affiliations:** 1 Paediatric Intensive Care Unit, Great Ormond Street Hospital, London, United Kingdom.; 2 Infection, Immunity and Inflammation Research and Teaching Department, University College London Great Ormond Street Institute of Child Health, London, United Kingdom.; 3 Children’s Acute Transport Service, Great Ormond Street Hospital for Children NHS Foundation Trust, London, United Kingdom.; 4 Clinical Trials Unit, Intensive Care National Audit & Research Centre, London, United Kingdom.; 5 Section of Anaesthetics, Pain Medicine and Intensive Care, Department of Surgery and Cancer, Faculty of Medicine, Imperial College London, London, United Kingdom.; 6 Cardiac Intensive Care Unit, Great Ormond Street Hospital, London, United Kingdom.

**Keywords:** Bayesian, clinical research, methodology, research networks, trials

## Abstract

**OBJECTIVES:**

To describe the rationale, design, and implementation of the Paediatric Intensive Care Adaptive Platform Trial (PIVOTAL), a novel approach to embed adaptive clinical research within routine pediatric critical care practice.

**DESIGN:**

Prospective, multicenter, Bayesian adaptive platform trial developed by the U.K. Paediatric Critical Care Society Study Group (PCCS-SG) and funded by the U.K. National Institute for Health and Care Research Health Technology Assessment program.

**SETTING:**

A minimum of 20 of the 33 PICUs across the United Kingdom that participating in the PCCS-SG research network.

**PATIENTS:**

Critically ill children admitted to a PICU requiring support for one or more organ systems.

**INTERVENTIONS:**

Eligible patients will be randomized across multiple concurrent intervention domains. Each domain will test clinically relevant therapies where practice variation and evidence gaps exist. The adaptive Bayesian design allows dynamic modification of randomization ratios, dropping of inferior interventions, and addition of new domains over time.

**MEASUREMENTS AND MAIN RESULTS:**

The primary outcome, common to all domains, is days alive and free of organ support to day 30. Trial efficiency and inclusivity are enhanced using routinely collected national registry data from the Paediatric Intensive Care Audit Network, real-time electronic health record integration, and a proportionate consent model. Extensive statistical simulations will inform decision rules for superiority, futility, and equivalence.

**CONCLUSIONS:**

PIVOTAL aims to bridge the gap between clinical care and research, providing a sustainable infrastructure to deliver timely, inclusive, and practice-changing evidence in pediatric critical care. Recruitment started in March 2026.

**RESEARCH IN CONTEXT**

- Randomized trials in pediatric critical care often take many years to complete, use temporary trial infrastructures, and typically address single questions that may become outdated before results are available.
- Adaptive platform trials potentially provide a more efficient model by evaluating multiple interventions simultaneously and adapting as evidence accumulates, exemplified by the COVID-19 studies in the Randomized Evaluation of COVID-19 Therapy (RECOVERY) and Randomized, Embedded, Multifactorial, Adaptive Platform Trial for Community-Acquired Pneumonia (REMAP-CAP) trials.
- The Paediatric Intensive Care Adaptive Platform Trial (PIVOTAL), developed by the U.K. Paediatric Critical Care Society Study Group, aims to embed Bayesian adaptive research within routine PICU care, using shared outcomes and national registry data with the aim of to generate faster, more inclusive, and clinically relevant evidence.

**AT THE BEDSIDE**

- PIVOTAL integrates randomized research into routine pediatric intensive care by enrolling eligible critically ill children and randomizing them across multiple treatment domains addressing common areas of clinical uncertainty.
- Initial domains evaluate fluid management, sedative choice, and RBC transfusion thresholds in mechanically ventilated patients, using a shared patient-centered outcome of days alive and free of organ support to day 30.
- Bayesian adaptive analyses allow treatments to be modified, dropped, or added as evidence accumulates, while integration with routinely collected data from the Paediatric Intensive Care Audit Network supports efficient recruitment and rapid translation of results into practice.

Randomized clinical trials (RCTs) are difficult, expensive and time consuming. Our experience in the U.K. Paediatric Critical Care Society Study Group (PCCS-SG) is that from initial idea through to primary publication of RCT results can take 10 years of coordinated work (1–6). Such a timeline is a major impediment to medical progress. By way of response to this predicament—not just in pediatric critical care research but in all RCTs—in 2024, Angus et al (7) wrote about the necessity for repairing “a house divided” (i.e., clinical practice and clinical research) and promoting greater integration between these two elements. Central to this Special Communication in the Journal of the American Medical Association is the observation that traditional clinical research often does not address the true areas of clinical uncertainty we face at the bedside, noting the transient exceptions in some healthcare systems during the COVID-19 pandemic. A further problem in the field of pediatric critical care or emergency medicine research is the challenge presented by consent processes that nudge us toward exclusion of less advantaged patients and families (8). That is, we need RCTs that include the whole population at risk to understand fully benefits or harms in the real world of bedside critical care.

The traditional model for a RCT that we and others around the world have used in pediatric critical care research has been to build “single-use” teams (1, 9–12). For example, we create case-report forms and develop randomization processes, we seek research ethics approval, and site approvals, all to reduce uncertainty. However, this process takes so long while practice is constantly evolving, that a result has the risk of being irrelevant by the time it is available. A newer approach—adaptive clinical trials—has potential to mitigate many of these weaknesses (13–15).

## PLATFORM TRIALS

The Randomized Evaluation of COVID-19 Therapy (RECOVERY) and Randomized, Embedded, Multifactorial, Adaptive Platform Trial for Community-Acquired Pneumonia (REMAP-CAP) trials demonstrated during the COVID-19 pandemic that multiple interventions can be studied simultaneously and at scale to provide answers in months rather than years during acute critical illness (15). These trials are “platforms” of a common infrastructure that is used to make multiple comparisons, which are called “domains” within the trial. This design can be combined with a Bayesian statistical approach to use information more efficiently as it becomes available, rather than after a preset sample size is met (16). The study can “adapt” to this new information in several ways. Randomization ratios might be altered to favor the better performing arm of the study, or the data may demonstrate that one arm is better (or worse, or equivalent) to another. This arm can then be “dropped,” and the platform used to incorporate other important questions (13).

In January 2025, the U.K. PCCS-SG commenced our latest project: the Paediatric Intensive Care Adaptive Platform Trial (PIVOTAL), which is funded by the U.K. National Institute for Health and Care Research Health Technology Assessment program.

## PAEDIATRIC INTENSIVE CARE ADAPTIVE PLATFORM TRIAL

The PIVOTAL is designed to close the gap between clinical practice and clinical research using the adaptive platform methodology (see https://www.icnarc.org/research-studies/pivotal/). The trial will enroll onto the platform patients admitted to the PICU who are receiving support for one or more organs. These patients will be randomized, initially, into up to three domains (a therapy area relating to one or more research questions) simultaneously. We are building on elements present in our recent trials, crucial to what we hope will complement this efficient design: use of routinely collected national registry data (Paediatric Intensive Care Audit Network [PICANet]) and a proportionate approach to information and consent (including a deferred consent model), which we have repeatedly shown is strongly supported by past-patients and their families (3, 8, 17–19). There are also novel elements to PIVOTAL that we have not employed previously: a common patient-centered primary outcome across all domains (days alive and free of organ support up to day 30); recruitment of postoperative cardiac patients in a separate analysis “stratum”; and the Bayesian adaptive approach to analysis as recruitment progresses, with a priori rules for declaring superiority, equivalence, or noninferiority of interventions within a domain.

**Figure 1.**
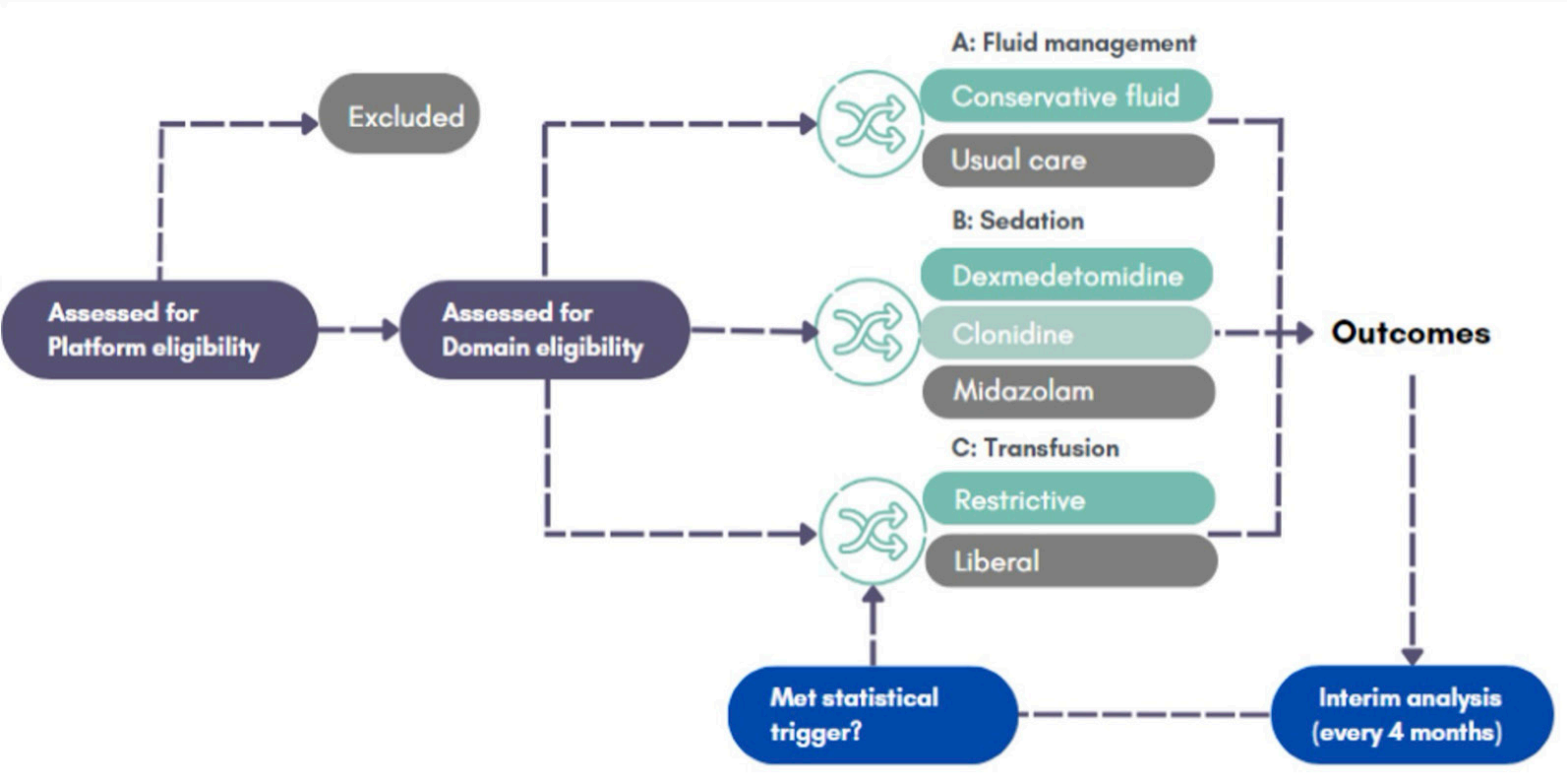
Overview of the Paediatric Intensive Care Adaptive Platform Trial (PIVOTAL).

The PIVOTAL program of work is based on an 11-month development grant, which included: a national research prioritization exercise involving clinicians, patients, and families; consultations on the governance structure and proposed methodology with clinicians, patients, and families (20); and intensive data and statistical modeling work to determine design and feasibility. As a result of this pilot work, we have selected three interventions that are: a) relevant to a high proportion of patients; b) show variation in care; and c) lack evidence for use (**Fig. 1** and **Table 1**). At the start, the three interventions are focused on mechanically ventilated patients and the use of fluids, sedatives and blood (**Table 2**). We estimate that we will recruit up to 320 patients per month from 20 our 33 national PICUs, which will provide a highly representative sample of the units in the United Kingdom. The adaptive approach means that sample sizes are not confirmed in advance but will likely be in the range of 8–12,000 critically ill children across the 5 years of funding (2026–2030).

**TABLE 1.** Key Terms in Adaptive Platform Trials and Their Application in PIVOTAL (Paediatric Intensive Care Adaptive Platform Trial)

Term	Definition	Application in PIVOTAL
Adaptive platform trial	A trial that studies multiple interventions simultaneously, with flexibility to adapt over time	PIVOTAL studies sedation, fluid, transfusion domains within one framework
Master protocol	Central trial framework covering shared elements; arms can be added via appendices	PIVOTAL’s protocol supports uniform conduct across domains
Domain	A clinical question or therapeutic area within the platform	PIVOTAL’s domains: sedation, fluid management, transfusion thresholds
Arm	A specific intervention or comparator within a domain	E.g., different sedative agents or transfusion thresholds
Shared control group	A common comparator for multiple arms to increase efficiency	Control children may serve as comparator across domains
Interim analysis	Preplanned data reviews to guide trial adaptation	Used to drop or promote interventions based on accruing outcomes
Response-adaptive randomization	Allocation ratios adjusted based on interim efficacy signals	May be implemented to favor effective arms
Bayesian methods	Statistical framework updating probabilities in real time	Considered for decision-making in interim analyses

**TABLE 2.** Table of Main Platform and Domain Level Inclusion and Exclusion Criteria, Key Characteristics of Interventions and Comparisons and Shared Platform Outcomes

Platform eligibility:			
Age > 37 wk corrected gestational age to < 16 yr			
Face to face with PICU staff or transport team			
Receiving support to at least one of respiratory, cardiovascular, or renal systems			
	**Domain A**	**Domain B**	**Domain C**
Population	I: Receiving invasive mechanical ventilation	I: Receiving invasive mechanical ventilation	I: Hemoglobin < 85 g/L (noncardiac) or < 100 g/L (cardiac non-neonate) or < 120 g/L (cardiac neonate)
	Within 12 hr of meeting platform criteria	Within 12 hr of meeting platform criteria	E: Hemoglobinopathies
	E: Admitted with a condition that requires a specific fluid management regimen (e.g., diabetic ketoacidosis)	Receiving, or about to receive, a continuous IV sedative	Cyanotic congenital heart disease
	Noncardiac stratum only	E: Known hypersensitivity or contraindication to one of the sedative agents	Receipt of RBC transfusion since meeting the platform inclusion criteria
		Noncardiac and cardiac strata	On extracorporeal membrane oxygenation
			Noncardiac and cardiac strata
Intervention(s)	Conservative fluid administration until the end of day 3—total daily fluid limited to a maximum of 60% (maximum 1.5 L/d) of the Holliday-Segar formula value^a^	Continuous IV infusion of dexmedetomidine	Restrictive transfusion strategy
	Active fluid removal—commenced D2 if there is evidence of fluid accumulation with cardiovascular stability	Continuous IV infusion of clonidine	Noncardiac: Hemoglobin of 65 g/L (70 g/L if CVI
			Cardiac: Hemoglobin of 70 g/L (80 g/L CVI), neonates 90 g/L (110 g/L CVI)
Comparator	Usual fluid management	Continuous IV infusion of midazolam	Liberal transfusion strategy
			Noncardiac: Hemoglobin 80 g/L (85 g/L CVI)
			Cardiac: Hemoglobin 80 g/L (100 g/L CVI), for neonates 100 g/L (120 g/L CVI)
Outcome	Days alive and free from organ failure to day 30		

CVI = cardiovascular instability.

a Holliday-Segar refers to the calculation of maintenance fluids at 100 mLs/kg for the first 10 kg of weight, 50 mLs/kg for additional weight up to 20 kg, and 20 mLs/kg for additional weight above 20 kg.

Note that full descriptions of the interventions are beyond the scope of this report.

### Statistical Modeling

To inform the design of PIVOTAL and ensure it has good operating characteristics, we conducted an iterative simulation exercise focusing on the initial research questions. The aim was to develop the statistical models and related processes, including statistical rules for triggering platform conclusions (i.e., superiority, inferiority, equivalence, or noninferiority). The output from each set of simulations was presented to and discussed by the PIVOTAL investigators to determine whether the design was satisfactory, and if not, where improvements should be made. The simulation setup was then modified accordingly, and further realizations of PIVOTAL simulated.

Each set of simulations required inputs defining the initial platform trial dimensions. That is: the primary clinical outcome; the patient population; enrollment and randomization rates; the statistical model; and statistical triggers. Additionally, scenarios were developed to test the proposed design under a range of possible “truths” including all interventions being equivalent and minimum clinically important differences (MCIDs). To inform the inputs, we drew on relevant data from PICANet, supplemented by recently completed clinical trials and observational studies.

Key to the design is a Bayesian regression model for the primary outcome that enables the evaluation of multiple interventions simultaneously, while allowing for the complexities in the collected data. This model is evaluated at regular intervals, and its output is used to determine whether the criteria for triggering any trial efficacy conclusion have been met. As the trial progresses and there are changes to the interventions and domains, the model will also evolve. A simplified version of the analysis model is used for simulation purposes.

The statistical triggers are domain specific, defined as a combination of posterior probabilities and numbers of recruited patients, and only evaluated when minimum numbers of patients have been randomized to the relevant interventions. In the simulated realizations, there is an automatic trial adaption if a trigger is achieved, but in practice, the Data Monitoring Committee (DMC) will have discretion about whether to make recommendations using the triggers as a guide. Considerations will include richer information provided to assist decision-making.

It is crucial that the design is robust to all plausible “truths,” and our test scenarios have been developed with clinical input to cover a wide range of possibilities, some more likely than others. This includes no, one and two interventions superior, different timings of effects, and more challenging situations such as borderline superiority (half MCID) or interactions between interventions in different domains. For each scenario and research question, we agreed, a priori, the minimum acceptability criteria in terms of the simulated probability of making correct decisions.

A wide range of tabular and graphical outputs have been produced from the simulations. For each domain, these include the probability of reaching correct and incorrect platform conclusions compared with our acceptability criteria, the timing of conclusions in months from recruitment start and in numbers of randomized patients, and platform sample sizes.

During the actual trial, there will be two types of analysis: adaptive and final. Adaptive analyses will be performed regularly to evaluate whether any statistical triggers that may lead to a platform conclusion have been met. A final analysis will follow each confirmation of a platform conclusion by the DMC, as specified in a domain-specific statistical analysis plan, encompassing all endpoints, subgroups, and secondary and sensitivity analyses. All modeling will use a Bayesian framework, with prior sensitivity explored and reported.

### Trial Governance and Capacity Building

**Figure 2.**
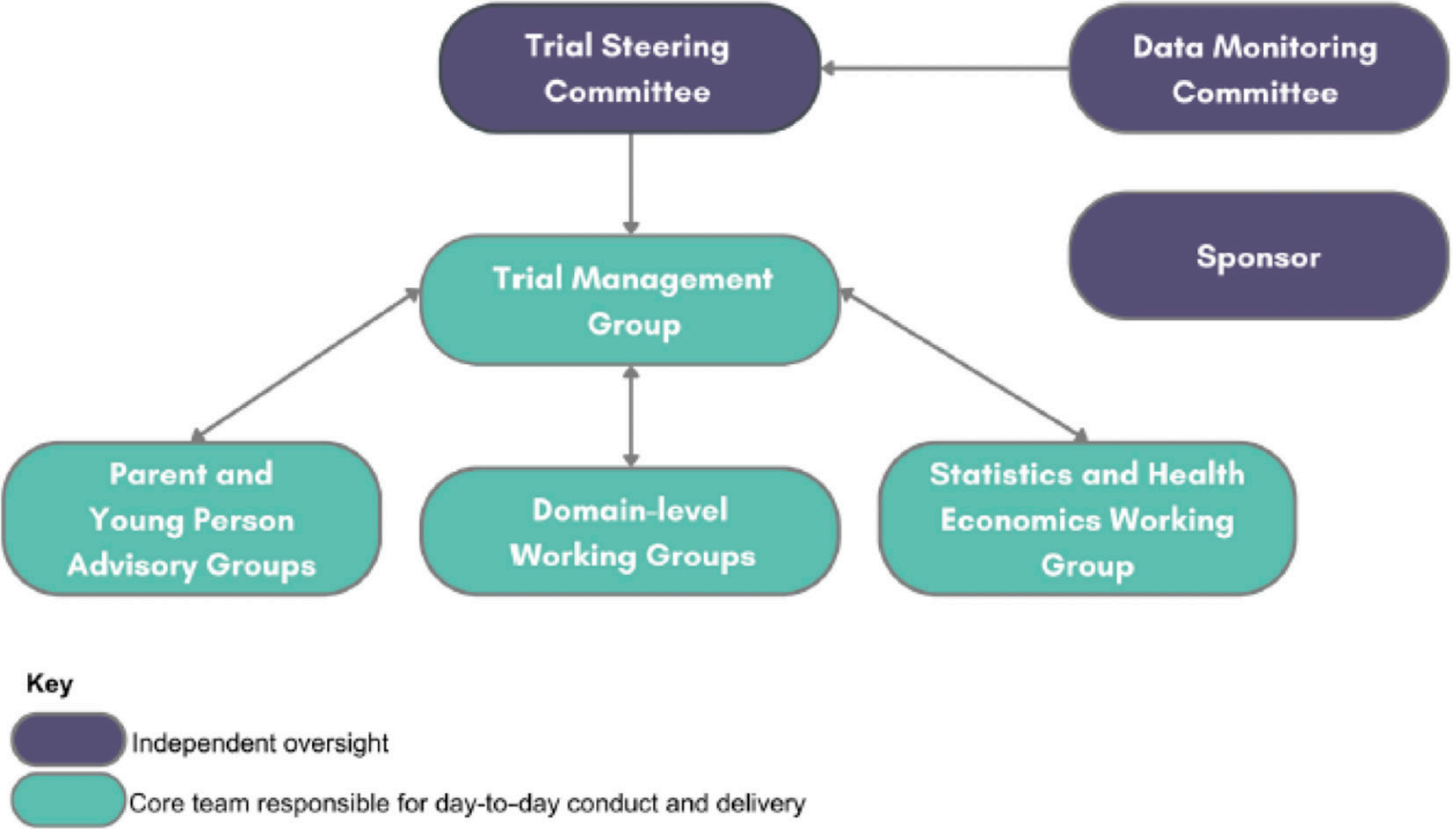
Paediatric Intensive Care Adaptive Platform Trial (PIVOTAL) governance organogram.

The governance structure of PIVOTAL has been designed with stakeholder input to provide appropriate management of all aspects of the Platform, considering representation of key stakeholders, expertise related to trial delivery and statistical analysis, and the prioritization of new research questions and domains (**Fig. 2**). The responsibility for the delivery of the platform rests with the Trial Management Group, with domains being actively managed by domain-specific working groups. To ensure the platform is being used to build capacity within the PICU research community, each domain is being co-led by an experienced investigator, alongside an early career investigator aiming to obtain experience of leading a large-scale clinical trial. Independent oversight will be provided by the Trial Steering Committee and DMC—both of which will oversee the conduct of all active domains within the platform.

As an adaptive platform, PIVOTAL is designed to continue beyond the initial domains. Inclusion of future domains will need three key aspects: 1) to be deemed a priority research question; 2) to be deemed as methodologically compatible with inclusion in the platform; and 3) to have adequate funding to be deliverable. Feedback on these aspects will be sought from key stakeholders including PCCS-SG, an independent Scientific Advisory Group and parents and young people. New domains will be included as amendments to the platform with no effect on already recruiting domains.

### Maximizing the Utility of Data

Ensuring that we minimize the burden of site research staff when collecting data for research use, we have aimed to use routinely collected data. In our U.K. PICUs, this includes the PICANet national clinical audit data and the National Health Service in England data for mortality and hospital episode statistics. This approach has been vital to the successful delivery of large-scale multicenter trials in U.K. PICUs (4, 21–23). To further extend this approach, PIVOTAL aims to explore the possibility of extracting data directly from electronic health records using an Application Programming Interface to automatically upload data into the PIVOTAL online secure database. This approach will not only reduce the burden of data collection for the research staff but also allow real-time collection of more granular data to understand the delivery of the interventions being studied and the wider contextual clinical factors.

Using data not only to maximize efficiency of data collection but also to understand operational aspects were deemed important by clinicians, patients, and families. PIVOTAL is aiming to do this in two important ways: to identify inclusivity of underserved populations throughout the recruitment and retention process and to understand whether research findings generated by the platform are being integrated into practice. With regards to inclusivity, approval has been sought to combine screening data on patients not randomized and PICANet data. Screening data will include the reason for nonrandomization, which could include factors such as the clinical decision not to randomize, missed by the research team, or a refusal of consent. This screening will enable identification of barriers to research inclusion and proactive actions to reduce these barriers enhancing inclusivity.

### Biobanking

Understanding biological mechanisms by which interventions lead to trial findings is a key priority for pragmatic clinical trials. As part of PIVOTAL, biological samples (e.g., whole blood for RNA sequencing, serum, and plasma) will be collected at the time of randomization at a subset of participating centers and stored for future analyses. Sampling will coincide with a routine blood draw from existing indwelling lines; since this will usually occur in an emergency, consent for biological sample storage and analysis will be obtained as part of the overall trial consent process. Samples will be discarded if consent is refused, as per our previous 2014–2016 biomarker study (24). Mechanistic studies will aim to understand the disease process, host response, and/or the differential effect of treatment regimens on the development and/or resolution of critical illness.

### Platform Proliferation and Harmonization

The wider critical care community for adult patients has adopted platform methodologies enthusiastically. Several large collaborations are now established and include clinical projects such as: Platform of Randomized Adaptive Clinical Trials in Critical Illness (PRACTICAL) with a focus on interventions for acute hypoxemic respiratory failure, starting with respiratory support and corticosteroids (see https://practicalplatform.org); and, Intensive Care Platform Trial (INCEPT) led from Denmark and examining dosing strategies for prophylactic low-molecular-weight heparin, use of albumin in shock, and continuous glucose monitoring (see https://incept.dk). There are also platforms that are aiming to use biological phenotyping to target interventions to those more likely to benefit. These include: Precision Medicine Adaptive Platform Network Trial in Hypoxemic Acute Respiratory Failure (PANTHER), a phase II trial aiming to examine if targeting medicines at hyperinflammatory and hypoinflammatory phenotypes of acute respiratory distress syndrome identified in real-time improves outcomes (see https://panthertrial.org) and time critical precision medicine for acute critical illness using treatable trait principles (TRAITS; see https://traits-trial.ed.ac.uk).

In pediatric critical care, the Pediatric Influence of Cooling Duration on Efficacy in Cardiac Arrest Patients (P-ICECAP) trial is recruiting using Bayesian adaptive methodology to address complex questions about timing and dose for therapeutic hypothermia after cardiac arrest (14). Several of the established PICU and emergency medicine research networks are working toward similar platforms (25). This includes the development of the Australia and New Zealand Intensive Care Society led Paediatric Intensive Care Adaptive Platform Trial (PLATINUM) in the PICU and the emergency medicine focused Paediatric Adaptive Sepsis Platform Trial (PASSPORT), which offer the opportunity to harmonize key aspects, such as population, research questions, and data collection specifications. These studies will allow the potential for federation of platform trials to answer important questions globally and for interventions that affect smaller groups of patients.

## CONCLUSIONS

Adaptive platform trials have potential advantages over traditional RCTs in answering individual questions. The PIVOTAL trial started recruitment in March 2026, with the immediate aims being to setup the infrastructure and address the three initially prioritized domain-specific questions. The longer-term goal is to fully embed the platform more deeply into routine clinical practice on PICUs by seamlessly answering questions and incorporating results into clinical practice. We are enthusiastic about making research part of routine practice in our PICUs. Reducing uncertainty in the questions we face at the bedside every day may follow. If we can demonstrate some of the efficiencies we hope for, the next challenge will be to build mechanisms to understand how best to target treatments at those who can benefit the most from them.

## APPENDIX

The Paediatric Intensive Care Adaptive Platform Trial (PIVOTAL) Investigators and participating sites as of December 2025: Domain Leads: A (Fluid)–Lyvonne Tume and Angela Aramburo; B (Sedation)–Rebecca Mitting; and C (Transfusion)–Avishay Sarfatti, Samiran Ray, and Simon Stanworth, Nazima Pathan, Esther Daubney (Addenbrooke’s Hospital); Gerri Sefton, Rachel Dewey (Alder Hey Children’s Hospital); Arun Ghose, Sarah Fox (Birmingham Children’s Hospital); Peter Davis, Elizabeth Stoddart, Judit Bak, Libby Greenberg (Bristol Royal Hospital for Children); Benjamin Crulli, Paul Wellman (Evelina London Children’s Hospital); Emma Simpson, Martin Cowie (Freeman Hospital); Elisabeth Day, Nadine Heenan (Great North Children’s Hospital); Katherine Brown, Samiran Ray, Mae Johnson, Thomas Brick, Kate Plant, Sarah Benkenstein, Rajan Sani (Great Ormond Street Hospital); Avishay Sarfatti, Melanie James (John Radcliffe Hospital); Akash Deep, Katie Tupper (King’s College Hospital); Jo Lumsden, Amisha Mistry (Leeds Children’s Hospital); Bedangshu Saikia, Laura Alcantara-Gemar (Leicester Royal Infirmary); Catarina Silvestre, Helen Bolam (Nottingham Children’s Hospital); Julie Richardson (Royal Belfast Hospital for Sick Children); Angela Aramburo (Royal Brompton Hospital); TBC (Royal Hospital for Children and Young People, Edinburgh); Elizabeth Henderson, Fiona Bowman (Royal Hospital for Children, Glasgow); Ramzi Hamzeh, Tahmina Khatun (Royal London Hospital); Ravishankar Nagaraj, Rebecca Marshall (Royal Manchester Children’s Hospital); Ahmed Sherif, Jack Watson (Royal Stoke University Hospital); Sandeep Budhiraja, Peter Kibe (Sheffield Children’s Hospital); John Pappachan, Amber Cook (Southampton Children’s Hospital); Buvana Dwarakanathan, Cristina DaSilva (St. George’s Hospital); and Toranj Wadia, Farhana Mehar (St. Mary’s Hospital).
